# A Method for Predicting the Creep Rupture Life of Small-Sample Materials Based on Parametric Models and Machine Learning Models

**DOI:** 10.3390/ma16206804

**Published:** 2023-10-22

**Authors:** Xu Zhang, Jianyao Yao, Yulin Wu, Xuyang Liu, Changyin Wang, Hao Liu

**Affiliations:** College of Aerospace Engineering, Chongqing University, Chongqing 400044, China

**Keywords:** small sample, creep rupture life prediction, time–temperature parametric models, machine learning models, comparison of model prediction accuracy

## Abstract

In view of the differences in the applicability and prediction ability of different creep rupture life prediction models, we propose a creep rupture life prediction method in this paper. Various time–temperature parametric models, machine learning models, and a new method combining time–temperature parametric models with machine learning models are used to predict the creep rupture life of a small-sample material. The prediction accuracy of each model is quantitatively compared using model evaluation indicators (RMSE, MAPE, R^2^), and the output values of the most accurate model are used as the output values of the prediction method. The prediction method not only improves the applicability and accuracy of creep rupture life predictions but also quantifies the influence of each input variable on creep rupture life through the machine learning model. A new method is proposed in order to effectively take advantage of both advanced machine learning models and classical time–temperature parametric models. Parametric equations of creep rupture life, stress, and temperature are obtained using different time–temperature parametric models; then, creep rupture life data, obtained via equations under other temperature and stress conditions, are used to expand the training set data of different machine learning models. By expanding the data of different intervals, the problem of the low accuracy of the machine learning model for the small-sample material is solved.

## 1. Introduction

The creep behavior of materials is of great concern to engineers when designing and evaluating materials for use in high-stress or high-temperature environments [1,2,3,4]. The term ‘creep’ describes a phenomenon in which, under certain temperature and stress conditions, a material slowly undergoes plastic deformation over time [5]. When material is in a high-temperature environment, the creep phenomenon is more obvious. Unlike brittle fracture, creep does not occur suddenly under the action of stress; on the contrary, strain accumulates slowly under long-term stress action. With the continuous development of the material creep process, excessive plastic deformation will occur in material components, leading to the failure of and damage to components, and even serious accidents [6,7]. Creep fracture is one of the principal failure modes of turbine blades in high-temperature environments. Creep leads to excessive plastic deformation of the blade and causes fracturing [8,9]. When the reactor cooling system of a nuclear power plant is heated up and pressurized, the excessively high temperature and pressure may cause creep failure in some positions of the steam generator heat transfer tube, leading the heat transfer tube to rupture. Such occurrences lead to the leakage of radioactive substances from the containment vessel and cause serious accidents [10,11,12,13]. In general, the occurrence of creep is gradual, and its outcome is always destructive. The prediction of the creep rupture life of materials is a significant problem in the field of engineering safety, and it is urgent to improve the reliability and accuracy of the prediction of material creep rupture life.

The Larson–Miller model is a time–temperature parametric model based on data fitting. The Larson–Miller model is often used in engineering to predict the creep rupture life of materials [14,15,16,17,18,19,20,21,22,23]. Recently, some researchers used machine learning models to predict the creep rupture life of materials [24,25,26,27,28,29]. Both the time–temperature parametric model and the machine learning model possess unique advantages when predicting material creep rupture life.

Due to the Larson–Miller parametric model being simple, easy to use, and having high prediction accuracy, it has attracted the attention of many researchers. Some researchers have used the Larson–Miller parametric model to study the creep properties and high-temperature creep behavior of various alloys. Kim et al. [14] predicted the long-term creep life of Gr.91 steel using the Larson–Miller parametric model and carried out reliability assessments. Niu et al. [15] developed a model for predicting the creep failure time and failure probability of heat transfer tube materials in nuclear power plants based on the Larson–Miller parametric model in order to study the risk of accidents potentially arising from high-temperature creep and improve the tube material’s ability to deal with serious accidents. Loghman et al. [16] calculated the creep damage of a thick-walled reactor made of 316 austenitic stainless steel using the Larson–Miller parametric model and evaluated its remaining life. Lee et al. [17] combined the data analysis method with the Larson–Miller parametric model to predict the creep rupture life of 2.25 Cr and 9~12% Cr ferritic steels. Pavan et al. [18] evaluated the creep rupture life of nickel-based superalloys from superheater coils in supercritical power plants using the Larson–Miller parametric model. Render et al. [19] predicted the creep rupture life of Inconel 740 alloy via the use of the Larson–Miller parametric model. Shi et al. [20] verified the high accuracy of the Larson–Miller parametric model in predicting the creep rupture life of various superalloys, including superalloys DD6, CMSX-4, CMSX-2, SC7-14-6, and Alloy-454. Cedro et al. [21] extrapolated the creep rupture life of Incoloy 800 alloy and 304H stainless steels using the Larson–Miller parametric model. Based on the experimental results of creep, Huang et al. [22] extrapolated creep rupture stress corresponding to the 100,000 h creep life of martensitic heat-resistant steel using the Larson–Miller parametric model, Monkman–Grant method, Norton power law, and creep damage tolerance. Sourabh et al. [23] predicted the creep rupture life of nickel-based 690 superalloys using the Larson–Miller parametric model. The authors further studied the high-temperature creep behavior of nickel-based 690 superalloys in a temperature ranging from 800 °C to 1000 °C.

Some researchers have tried to predict the creep rupture life of some alloys with the help of machine learning models, finding that some machine learning models have high prediction accuracy in terms of life prediction. Zhang et al. [24] predicted the creep fracture life of 316 austenitic stainless steel using machine learning models (Gaussian process regression model, random forest model, support vector machine model, and shallow neural network model) and a deep learning model (deep neural network model). Wang et al. [25] converted the creep data of Cr-Mo steel into Larson–Miller parameters and other time–temperature parameters, and then predicted the creep rupture life of Cr-Mo steel using different models: the linear regression model, random gradient descent model, multi-layer perceptron model, and support vector machine model. Tan et al. [26] proposed an integrated model coupled with Larson–Miller parameters and predicted a creep rupture life of 9% Cr martensitic heat-resistant steel through individual machine learning models (linear regression, support vector machine, and artificial neural network models) and integrated learning models, evaluating the prediction accuracy of each model. He et al. [27] predicted the creep fracture behavior of austenitic heat-resistant steel Sanicro 25 using a soft-constrained machine learning model. Xiang et al. [28] predicted the creep rupture life of Fe-Cr-Ni heat-resistant alloy using a deep learning model. Zhu et al. [29] predicted the properties of GH4169D alloy via comparison with GH4169 alloy. Further, the authors predicted the high-temperature creep rupture life using the low-temperature creep rupture life of GH4169 and GH4169D alloys. The prediction accuracy was higher than 90%.

In addition to predicting the creep rupture life of alloys via machine learning models, the researchers also used such methods to study the effects of factors related to the creep properties of alloys. Liu et al. [30] developed a divide-and-conquer self-adaptive (DCSA) machine learning model to take into account not only alloy composition, test temperature, and test stress, but also the microscopic structural parameters related to the creep process, e.g., layer-fault energy, lattice parameters, and diffusion coefficient. They predicted the creep rupture life of Ni-based single-crystal superalloys and investigated the effect of microstructure on the creep properties of Ni-based single-crystal superalloys. Kong et al. [31] optimized the machine learning model using a genetic algorithm. These authors then predicted the creep rupture life of 9% Cr alloy and studied the relationship between the composition and creep properties of 9% Cr alloy. Han et al. [32] predicted the creep rupture life of nickel-based single-crystal superalloys using machine learning models and studied the effects of different alloy elements on the creep life of nickel-based single-crystal superalloys. Khatavkar et al. [33] developed a large database of nickel-based superalloys, predicted the ultimate tensile strength, yield strength, and creep fracture life of nickel-based superalloys through machine learning models, and quantified the contribution of various characteristics to model prediction results through SHAP (Shapley additive explanations) values. Feng et al. [34] predicted the creep performance of recycled aggregate concrete through two types of models (individual machine learning and ensemble learning). Feng analyzed the importance of recycled aggregate concrete and studied the effects of different input variables on its creep performance based on the extreme gradient boosting model. Wang et al. [35] combined machine learning models with genetic algorithms to predict the creep rupture life of low-alloy steel, studying the effects of alloy composition and processing parameters on the creep properties of low-alloy steels for the design and development of new alloys.

These studies confirm the feasibility of using various machine learning models to predict the creep rupture life of certain materials. However, the results of the researchers’ predictions have not yet been compared with the prediction results of classical parametric models, which in fact on occasion have high accuracy in predicting the creep rupture life of materials. In this paper, the prediction accuracy of the Larson–Miller, Mason–Succop, Ge–Dorn, and Manson–Haferd parametric models and several common machine learning models are compared. The prediction ability of each model is evaluated quantitatively using three model evaluation indicators, and model selection is carried out to improve the accuracy of material creep rupture life prediction.

By converting the creep test data for fitting-related application to the P−lgσ coordinate system, the time–temperature parametric model can always obtain a curve prediction function with a high fitting degree in relation to the test data. The machine learning models can train creep test data through different algorithm theories, predict the creep rupture life under different conditions, and quantify the influence of different input variables on the output for comparison. Due to the different theories of various models in the two methods and their different applicability to varied materials, the prediction ability of each model for different types of creep data is always varied. The current research always focuses on using one of the two methods in prediction in order to find the model with the strongest prediction ability and apply it to the prediction of material creep rupture life. Researchers do not account for the different applicability of each model to a variety of materials. A model with a strong prediction ability for one material may not have similar utility when making forecasts about other materials. It is not guaranteed that a certain model always has the strongest prediction ability for a variety of materials.

In this paper, we propose a creep rupture life prediction method. Different models are used to predict the creep rupture life of materials, including several classical time–temperature parametric models and various machine learning models. Then, the prediction accuracy of each model is quantitatively compared using model evaluation indicators (RMSE, MAPE, R^2^), and the predicted result of the model with the strongest prediction accuracy is the output. This prediction method not only improves the applicability and accuracy of creep rupture life prediction but also quantifies the influence of each input variable on creep performance through the machine learning model.

The most common problem faced when predicting material creep rupture life with small-sample data via the use of machine learning models is the low prediction accuracy of machine learning models due to insufficient data in the training set. The amount of data in the training set is a decisive factor affecting the prediction accuracy of a machine learning model. The question of how to extend creep data reasonably and improve the prediction accuracy of material creep rupture life is an urgent problem in need of resolution. In this paper, a new method is proposed that combines the classical time–temperature parametric models with advanced machine learning models and gives full play to the advantages of the two methods. The parametric equation of creep life, stress, and temperature is obtained using different time–temperature parametric models, and then the creep life data of other conditions predicted via the equation are used to expand the training set data of different machine learning models. Through this method, the advanced machine learning model is combined with the classical time–temperature parametric model. This not only solves the problem that the machine learning model is difficult to use on small samples but also improves the prediction accuracy of the machine learning model.

## 2. Three Categories of Models Used in the Prediction Method

### 2.1. Time–Temperature Parametric Models

#### 2.1.1. Larson–Miller Parametric Model

**Figure 1:**
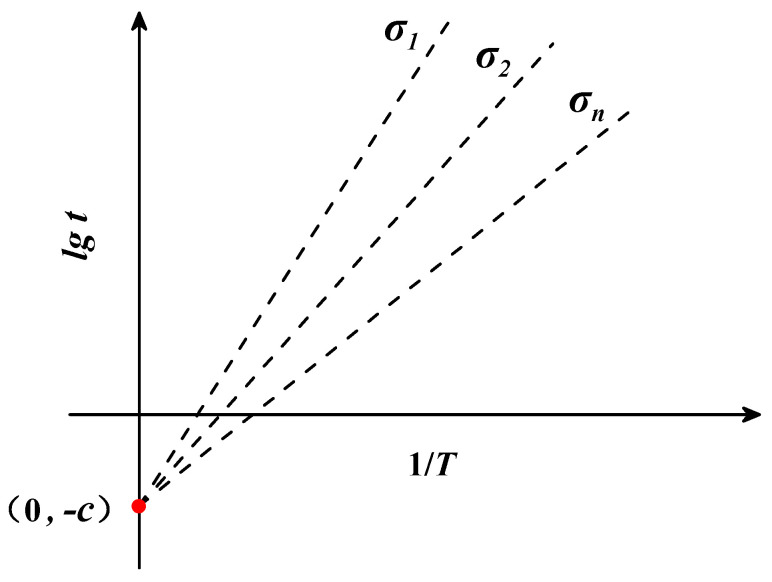
The theoretical diagram of the Larson−Miller parametric model.

In 1952, Larson and Miller [36] found that, under a certain level of stress, the logarithmic creep fracture time lgt of the material tends to be linear with the inverse of temperature 1/T. Based on this law, they proposed the Larson–Miller parametric model, in order to convert temperature T and logarithmic fracture time lgt into comprehensive parameter P. The comprehensive parameter P is composed of temperature T, fracture time t, and fitting parameter $c_{LM}$. In this way, the creep test data at different temperatures and stress conditions are converted into a series of points in a two-dimensional right-angle coordinate system (P − lgσ), and a cubic function curve can be obtained on the basis of fitting this series of points. Once the fitted curve is obtained, it can be used to predict the creep fracture time of the material under other temperature and stress conditions. The mathematical equations of the L-M model are as follows:

(1) $$P(\sigma )=T\cdot (c_{LM}+lgt)$$

(2) $$\mathrm{lg}\sigma =a_{0}+a_{1}\cdot P(\sigma )+a_{2}\cdot P^{2}(\sigma )+a_{3}\cdot P^{3}(\sigma )$$

where t is the fracture time (h), T is the temperature (K), $c_{LM}$ is a constant determined by creep test data, and P(σ) is a function of the stress σ. When the stress σ is certain, P(σ) is a definite value and the relationship between 1/T and lgt is linear. When a linear function passes through the fixed point (0, $-c_{LM}$), the value of the constant $c_{LM}$ can be obtained by solving for the intercept of the linear function. The relationship between lgt and 1/T is depicted in Figure 1.

#### 2.1.2. Manson–Succop Parametric Model

**Figure 2:**
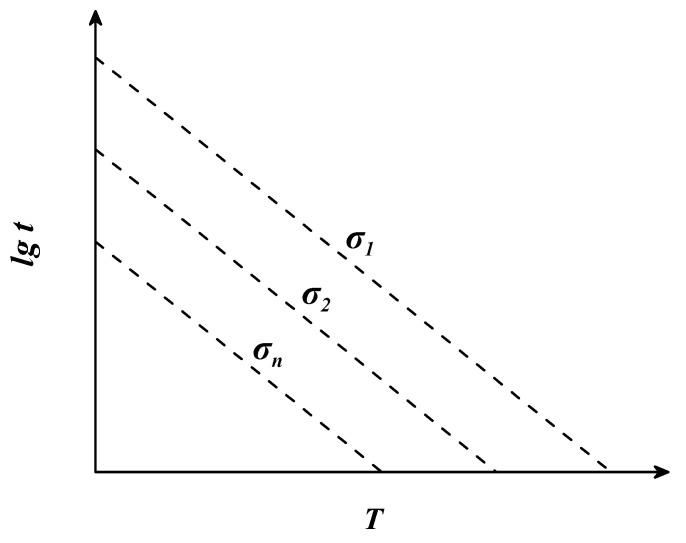
The theoretical diagram of the Manson–Succop parametric model.

Manson and Succop [37] found that, under certain stress conditions, the logarithmic creep fracture time lgt of the material tends to be linear with the temperature T. Based on this observation, they proposed the Manson–Succop parametric model, which converts temperature T and logarithmic fracture time lgt into the parameter P. Similar to the L-M model, the M-S model converts the creep test data at different temperatures and stress conditions into a series of points in a two-dimensional right-angle coordinate system (P − lgσ). A curve can be obtained by fitting this series of points, which is a cubic function. This curve is then used to predict the creep fracture time of the material under other temperature and stress conditions. The mathematical equations of the M-S model are as follows:

(3) $$P(\sigma )=\mathrm{lg}t-c_{MS}\cdot T$$

(4) $$lg\sigma =a_{0}+a_{1}\cdot P(\sigma )+a_{2}\cdot P^{2}(\sigma )+a_{3}\cdot P^{3}(\sigma )$$

where t is the fracture time (h), T is the temperature (K), $c_{MS}$ is a constant determined by creep test data, and $P\left(\sigma \right)$ is a function of the stress σ. When the stress is certain, $P\left(\sigma \right)$ is a definite value and the relationship between T and lgt is linear. The slope of linear functions under different stress conditions is represented by $c_{MS}$. Therefore, the constant $c_{MS}$ can be obtained by solving for the slope of a linear function. The relationship between lgt and *T* is shown in Figure 2.

#### 2.1.3. Ge–Dorn Parametric Model

The Ge–Dorn parametric model asserted that [38,39], under a certain level of stress, 1/T and lgt are linearly related, and the slope of linear functions under different stresses is $c_{GD}$. Therefore, the constant $c_{GD}$ can be obtained by solving for the slope of a linear function. The mathematical equations of the G-D model are as follows:

(5) $$P(\sigma )=\mathrm{lg}t-c_{GD}/T$$

(6) $$\mathrm{lg}\sigma =a_{0}+a_{1}\cdot P(\sigma )+a_{2}{\cdot P}^{2}(\sigma )+a_{3}\cdot P^{3}(\sigma )$$

where t is the fracture time (h), T is the temperature (K), $c_{GD}$ is a constant determined by creep test data, and $P\left(\sigma \right)$ is a function of the stress σ. When the stress is certain, $P\left(\sigma \right)$ is a definite value and the relationship between 1/T and lgt is linear. The slope of the linear functions under different stress conditions is $c_{GD}$. Therefore, the constant $c_{GD}$ can be obtained by solving for the slope of a linear function.

**Figure 3:**
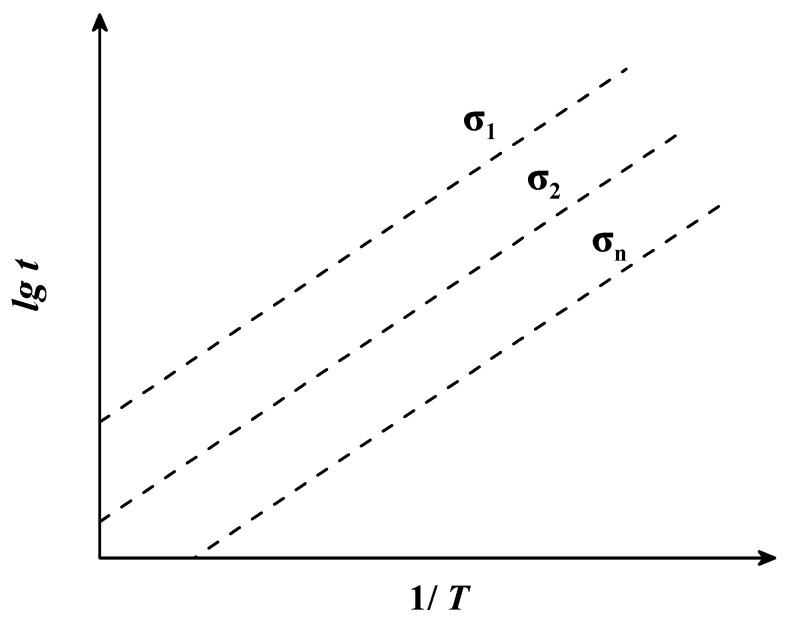
The theoretical diagram of the Ge–Dorn parametric model.

The relationship between lgt and 1/T is shown in Figure 3.

#### 2.1.4. Manson–Haferd Parametric Model

**Figure 4:**
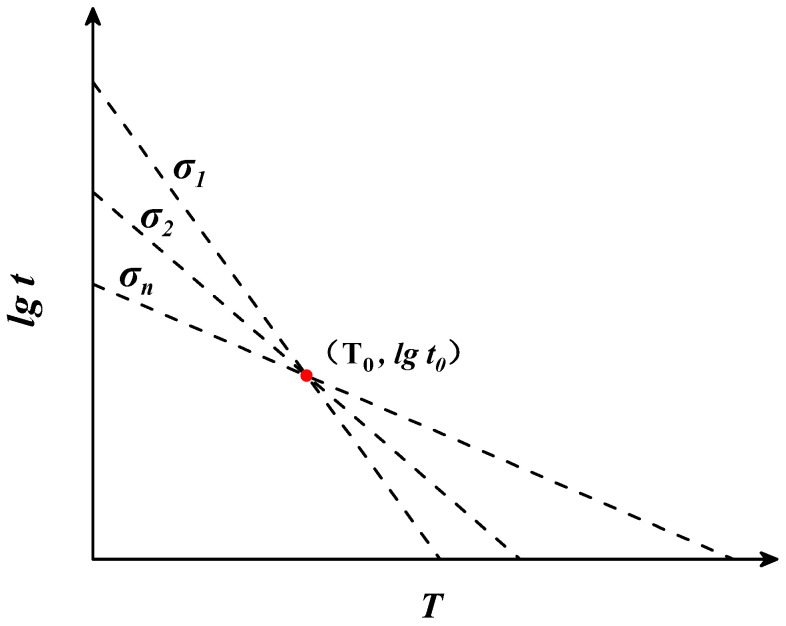
The theoretical diagram of the Manson–Haferd parametric model.

Manson and Haferd [40] found that, under a certain level of stress, there is a linear relationship between T and lgt. Their research also revealed that the linear function passes through the fixed point ${(T}_{0},lgt_{0})$. Based on this law, they proposed the Manson–Haferd parametric model, which converts temperature T and fracture time lgt into parameter P through their proposed equation. The mathematical equations of M-H model are as follows:

(7) $$P(\sigma )=\left(lgt-lgt_{0}\right)/\left(T-T_{0}\right)$$

(8) $$\mathrm{lg}\sigma =a_{0}+a_{1}\cdot P(\sigma )+a_{2}\cdot P^{2}(\sigma )+a_{3}\cdot P^{3}(\sigma )$$

where t is the fracture time (h), T is the temperature (K),$T_{0}$ and $lgt_{0}$ are constants determined by creep test data, and $P\left(\sigma \right)$ is a function of the stress σ. When the stress is certain, $P\left(\sigma \right)$ has a definite value and the relationship between $\left(T-T_{0}\right)$ and $\left(lgt-lgt_{0}\right)$ is linear. The values of two constants, namely, $lgt_{0}$ and $T_{0}$, can be obtained by finding the coordinates of the intersection of linear functions under different stress conditions. The relationship between lgt and T is shown in Figure 4.

### 2.2. Machine Learning Models

Some researchers have applied different machine learning models to the task of predicting the creep rupture life of certain materials, finding that some models possess strong prediction capacities [41,42,43]. Considering the different algorithm theories and applicability of different machine learning models, this paper adopts several common machine learning models to predict the creep rupture life of materials, comparing the results with those of other methods.

The following describes the basic prediction principles and limitations of the machine learning models used in this paper. The input variables of each machine learning model are the mass fraction of different elements, the test temperature T, and the test stress σ. Additionally, the output variable is the logarithmic creep rupture time lgt of the material. By far the largest difference between machine learning models is that they train the input data using different algorithmic theories.

#### 2.2.1. Back-Propagation Neural Network Based on Particle Swam Optimization (PSO-BPNN) [44,45]

The PSO-BPNN model uses a particle swarm optimization algorithm to optimize a back-propagation neural network (BPNN), adjust the weight and bias of the neural network, improve its training efficiency and accuracy, assist it in producing the optimal local solution and improve its global search ability. By constantly adjusting the neural network, the model can learn the complex relationship between data and obtain accurate prediction results.

The PSO-BPNN model is sensitive to the selection and preprocessing of input features. Indeed, inappropriate feature selection will lead to the underfitting or overfitting of the model, which in turn will affect the generalization ability of the model. Since the PSO-BPNN model involves the training of a BP model and the iterative optimization searching of a PSO algorithm, it requires a two-stage training process. Indeed, the training time required is longer than that needed for a BPNN-only method. Some parameters must be set manually in the PSO-BPNN model, including the number of particles, the number of iterations, the learning rate, etc. For different problems, some parameters must be adjusted in order to adapt the model to situational specifics.

#### 2.2.2. Back-Propagation Neural Network Based on Genetic Algorithms (GA-BPNN) [46,47,48]

The GA-BPNN model combines the genetic algorithm and the back-propagation neural network (BPNN) and uses the genetic algorithm to optimize the weight and threshold of the back-propagation neural network, overcoming the problem that back-propagation neural networks easily fall into the local optimal solution.

The GA-BPNN model is endowed with good application effect when applied to nonlinear problems and used to solve high-dimensional features. It can make full use of the global search characteristics of genetic algorithms and the prediction ability of back-propagation neural networks to achieve accurate prediction and strong generalization ability.

The GA-BPNN model has poor interpretability due to its complex network structure and the randomness of genetic algorithms. Similar to the limitations of the PSO-BPNN model, the GA-BPNN model involves genetic algorithm optimization and neural network model training, which takes a long time for large-scale data sets. The GA-BPNN model also has some parameters that need to be set manually, including population size, evolutionary algebra, crossover rate, mutation rate, etc.

#### 2.2.3. Radial Basis Function Neural Network (RBFNN) [49,50,51]

The RBFNN model is a three-layer neural network model composed of an input layer, a nonlinear hidden layer, and a linear output layer. The RBFNN model is characterized by local sensing ability and global approximation ability. By mapping the input sample to the hidden layer neuron, it uses the radial basis function to measure the similarity of the input sample, transforming it into a high-dimensional feature space for modeling.

The RBFNN model calculates the final output result according to the output of the hidden layer and the corresponding weight value. The RBFNN model has a strong generalization ability and fast convergence ability and is widely used in various fields.

Although the RBFNN model has a strong nonlinear mapping ability, its parameters are often difficult to explain. Furthermore, it is difficult to explain and understand the relationship between each hidden layer and its role.

#### 2.2.4. Random Forest (RF) [52,53,54]

An RF model is based on decision trees that use self-aggregation and random feature selection to reduce overfitting risk and improve model performance. An RF model averages or votes the predicted results of all decision trees to obtain the final predicted results. For the regression problem, the average value of a set of training samples is saved on the leaf nodes of each decision tree. When making a prediction, each decision tree provides a prediction result, and the final prediction result is the average of the predictions of all decision trees.

RF models can effectively reduce variance and overfitting risk by integrating prediction results from multiple decision trees. Since each decision tree is built independently, the model has strong noise resistance. RF models can also provide feature importance assessments to help analyze the degree to which a feature contributes. The model has high flexibility and robustness in practical application, being suitable for application to various data types and problems.

Although an RF model uses bootstrapping and the random selection of features to reduce overfitting, the model may still overfit if the sample size is too small or the correlation between features is too high. Because RF models comprise multiple decision trees, each trained on a set of randomly selected features, understanding and interpreting the entire model becomes a relatively complex task.

#### 2.2.5. Support Vector Regression (SVR) [55,56]

An SVR model maps the training data onto a high-dimensional feature space and searches for a hyperplane in the high-dimensional feature space. When the eigenvalue of a new sample is given, the model maps the sample onto a high-dimensional space and predicts according to the position of the sample on the hyperplane.

An SVR model controls the complexity of the model by introducing a penalty term, thus avoiding the problem of overfitting. SVR models can deal with linear and nonlinear regression problems and adapt to different data features by selecting different kernel functions. SVR models can effectively process high-dimensional data and sample noise. Additionally, they are endowed with strong robustness.

For complex problems with multiple variables, SVR models may not be able to effectively capture the characteristics of and relationships between the data. Indeed, when the data set is large or there are many input features, SVR models require a long period of training. In addition, SVR models are sensitive to noise. As such, in a high-noise environment, SVR models may not achieve such a good performance.

#### 2.2.6. Deep Neural Network (DNN) [57]

A DNN model is a nonlinear model that can adapt to complex data features and relationships. The model consists of multiple hidden layers, each containing multiple neurons. Each neuron is connected to all neurons in the previous layer, and an activation function is applied to each neuron. The activation function plays a role in weighting the input information and nonlinear transformation in the neural network.

A DNN model maps the input data to the corresponding output according to the combination of weight and bias and the action of the activation function. Through back-propagation and a process of updating parameters during the training process, parameters are optimized in order to improve the accuracy of the prediction results.

Due to the DNN model’s strong fitting ability, if the training data are insufficient or the training set and test set do not match, the model may overfit the training data, resulting in a poor performance in its application to the test set. DNN models are sensitive to noise and outliers in the data and are easily disturbed by them.

#### 2.2.7. Gauss Process Regression (GPR) [58,59]

A GPR model is a non-parametric Bayesian model designed for regression problems. The prediction principle of the model is to build a Gaussian process model for the target variables in the training data and to use the model to predict new input data.

The model assumes that the target variables obey a multivariate Gaussian distribution, obtaining similarity information between the target variables via the calculation of the covariance matrix between the training data.

When a new input sample is available, the distribution of the predicted values is inferred by calculating the covariance between that sample and the training data. The entire prediction process of the model is based on the principle of Bayesian inference. By optimizing the hyperparameters of the model, it may be adjusted adaptively to better fit the data and predict the target variables of unknown samples.

The computational complexity of a GPR model increases rapidly with the increase in data scale. Because a GPR model is used to calculate the inverse matrix of the covariance matrix, the calculation, and storage of the covariance matrix become more difficult with the increase in data dimension, and the sampling and interpolation of high-dimensional data also requires more computing resources, which may not meet the efficiency requirements.

#### 2.2.8. Deep Belief Network (DBN) [60,61]

A DBN model is a deep learning model that predicts by stacking multiple RBM (restricted Boltzmann machines) in order to construct a multi-layer neural network. During the training phase, a DBN model is built layer by layer through an approach centered around pre-training and fine-tuning. In the pre-training process, each layer’s RBM learns the distribution characteristics of the data. Then, the learned weights are used as inputs for the next layer’s RBM, gradually extracting features from higher-level representations.

In the fine-tuning phase, the entire network is adjusted using a back-propagation algorithm in order to minimize the prediction error on the training data. Through this hierarchical training approach, the DBN model can learn more abstract features at higher levels and has a strong non-linear modeling capability.

In a DBN model, when a gradient update is carried out using the back-propagation algorithm, the problem of gradient disappearance may occur. This leads to an unstable training process and makes the network unable to converge or difficult to optimize. When a DBN model has too many layers, it is easy for the gradient to disappear. In the process of back-propagation, the gradient will gradually become smaller with the increase in the number of layers. When the number of layers is too large, the gradient may become very small, making the network unable to update effectively.

### 2.3. A New Method of Predicting the Creep Rupture Life of Materials

#### 2.3.1. A Method Combined with the Parametric Models and the Machine Learning Models

For any material, new or old, there must be one similar, with roughly the same type of elements but a different content of elements. Although the chemical formulae of the two materials differ, the creep rupture life data of the two materials can be fused via machine learning models because the two materials share the same variables, such as temperature, stress, chemical elements, etc. However, this method has a serious shortcoming: due to the long time and high cost of performing high-temperature creep tests, very limited creep data are obtained through the test. As a result, the distribution of creep data in various data intervals is often unbalanced, and most of the data are concentrated in a certain interval. Although the sample size of a training set is expanded via the introduction of data from similar materials, the prediction accuracy of machine learning models is still not high enough.

In order to solve this problem, we propose a new prediction method that combines the classical time–temperature parametric models with advanced machine learning models and gives full play to the advantages of the two categories of methods. The specific idea is to reasonably expand the data in various intervals of the machine learning model training set using the time–temperature parametric model in order to balance the distribution of the data set in various intervals and further improve the applicability and prediction accuracy of machine learning models.

The four types of time–temperature parametric models used in the new method are the L-M model, M-S model, G-D model, and M-H model. The L-M, M-S, and G-D models combine temperature T with logarithmic creep fracture time lgt by applying constant c to equations related to logarithmic stress lgσ. The M-H model combines temperature T with the logarithmic creep fracture time lgt using two constants, $T_{0}$ and $lgt_{0}$, in order to form an equation related to logarithmic stress lgσ. The equations of the L-M, M-S, G-D, and M-H models are shown in Equations (9)–(12), respectively.

L-M:

(9) $$\mathrm{lg}\sigma =a_{0}+a_{1}\cdot T\left(c_{LM}+\mathrm{lg}t\right)+a_{2}\cdot T^{2}{\left(c_{LM}+\mathrm{lg}t\right)}^{2}+a_{3}\cdot T^{3}{\left(c_{LM}+\mathrm{lg}t\right)}^{3}\;$$

M-S:

(10) $$\mathrm{lg}\sigma =a_{0}+a_{1}\cdot \left(\mathrm{lg}t-c_{MS}\cdot T\right)+a_{2}\cdot {\left(\mathrm{lg}t-c_{MS}\cdot T\right)}^{2}+a_{3}\cdot {\left(\mathrm{lg}t-c_{MS}\cdot T\right)}^{3}\;$$

G-D:

(11) $$\mathrm{lg}\sigma =a_{0}+a_{1}\cdot \left(\mathrm{lg}t-\frac{c_{GD}}{T}\right)+a_{2}\cdot {\left(\mathrm{lg}t-\frac{c_{GD}}{T}\right)}^{2}+a_{3}\cdot {\left(\mathrm{lg}t-\frac{c_{GD}}{T}\right)}^{3}$$

M-H:

(12) $$\mathrm{lg}\sigma =a_{0}+a_{1}\cdot \left(\frac{lgt-lgt_{0}}{T-T_{0}}\right)+a_{2}\cdot {\left(\frac{lgt-lgt_{0}}{T-T_{0}}\right)}^{2}+a_{3}\cdot {\left(\frac{lgt-lgt_{0}}{T-T_{0}}\right)}^{3}$$

Equations (13)–(16) can be obtained by expanding Equations (9)–(12). We found from Equations (13)–(15) that, for L-M, M-S, and G-D models, if the values of the fitting coefficients $a_{3}$, $a_{2}$, $a_{1}$, and $a_{0}$ and the value of constant c are all determined, it is possible to obtain equations for logarithmic stress lgσ, logarithmic creep fracture time lgt and temperature T. It can be found from Equation (16) that for the M-H model, if the values of the fitting coefficients $a_{3}$, $a_{2}$, $a_{1}$, $a_{0}$ and the values of constants $T_{0}$ and $lgt_{0}$ are all determined, a cubic equation about logarithmic stress lgσ, logarithmic creep fracture time lgt and temperature T can be obtained. The equations of four models can be unified as Equation (17). For Equation (17), once the values of the logarithmic stress lgσ and temperature T are determined, the cubic equation of the logarithmic creep fracture time lgt can be obtained. By solving the roots of the cubic equation, the logarithmic creep fracture time lgt can be obtained. In this way, the creep fracture time values under other temperature and stress conditions can be predicted using known creep test data.

Four classical time–temperature parametric models are used to obtain parametric equations of temperature T, stress σ, and creep life t. Then, the creep fracture time data of other conditions obtained from parametric equations are expanded into the training set of machine learning models to realize the combination of the time–temperature parametric models and the machine learning models.

L-M:

(13) $$a_{3}\cdot T^{3}{\left(\mathrm{lg}t\right)}^{3}+3a_{3}c_{LM}\cdot T^{3}{\left(\mathrm{lg}t\right)}^{2}+a_{2}\cdot T^{2}{\left(\mathrm{lg}t\right)}^{2}+3a_{3}{c_{LM}}^{2}\cdot T^{3}\left(\mathrm{lg}t\right)+2a_{2}c_{LM}\cdot T^{2}\left(\mathrm{lg}t\right)+a_{3}{c_{LM}}^{3}\cdot T^{3}\;+a_{2}{c_{LM}}^{2}\cdot T^{2}+a_{1}\cdot T\left(\mathrm{lg}t\right)+a_{1}c_{LM}\cdot T+a_{0}-\mathrm{lg}\sigma =0$$

M-S:

(14) $$a_{3}\cdot {\left(\mathrm{lg}t\right)}^{3}-3a_{3}c_{MS}\cdot T{\left(\mathrm{lg}t\right)}^{2}+a_{2}\cdot {\left(\mathrm{lg}t\right)}^{2}+3a_{3}{c_{MS}}^{2}\cdot T^{2}\left(\mathrm{lg}t\right)-2a_{2}c_{MS}\cdot T\left(\mathrm{lg}t\right)+a_{1}\left(\mathrm{lg}t\right)-a_{3}{c_{MS}}^{3}\cdot T^{3}\;+a_{2}{c_{MS}}^{2}\cdot T^{2}-a_{1}c_{MS}\cdot T+a_{0}-\mathrm{lg}\sigma =0$$

G-D:

(15) $$a_{3}\cdot {\left(\mathrm{lg}t\right)}^{3}-\frac{3a_{3}c_{GD}}{T}{\left(\mathrm{lg}t\right)}^{2}+a_{2}\cdot {\left(\mathrm{lg}t\right)}^{2}+\frac{3a_{3}{c_{GD}}^{2}}{T^{2}}\left(\mathrm{lg}t\right)-\frac{2a_{2}c_{GD}}{T}\left(\mathrm{lg}t\right)+a_{1}\cdot \left(\mathrm{lg}t\right)-\frac{a_{3}{c_{GD}}^{3}}{T^{3}}+\frac{a_{2}{c_{GD}}^{2}}{T^{2}}\;-\frac{a_{1}c_{GD}}{T}+a_{0}-\mathrm{lg}\sigma =0$$

M-H:

(16) $$\frac{a_{3}}{{\left(T-T_{0}\right)}^{3}}\cdot {\left(\mathrm{lg}t\right)}^{3}-\frac{{3a}_{3}lgt_{0}}{{\left(T-T_{0}\right)}^{3}}\cdot {\left(\mathrm{lg}t\right)}^{2}+\frac{a_{2}}{{\left(T-T_{0}\right)}^{2}}\cdot {\left(\mathrm{lg}t\right)}^{2}-\frac{2a_{2}lgt_{0}}{{\left(T-T_{0}\right)}^{2}}\cdot \mathrm{lg}t+\frac{3a_{3}{\left(lgt_{0}\right)}^{2}}{{\left(T-T_{0}\right)}^{3}}\cdot \left(\mathrm{lg}t\right)+\frac{a_{1}}{T-T_{0}}\cdot \mathrm{lg}t\;-\frac{a_{3}}{{\left(T-T_{0}\right)}^{3}}\cdot {\left(lgt_{0}\right)}^{3}+\frac{a_{2}}{{\left(T-T_{0}\right)}^{2}}\cdot {\left(lgt_{0}\right)}^{2}-\frac{a_{1}}{T-T_{0}}\cdot \left(lgt_{0}\right)+a_{0}-\mathrm{lg}\sigma =0$$

(17) $$f\left(\mathrm{lg}\sigma ,T-T_{0},\mathrm{lg}t-lgt_{0}\right)=0,\left(LM,MS,GD:T_{0}=0\;and\;lgt_{0}=0\right)$$

**Figure 5:**
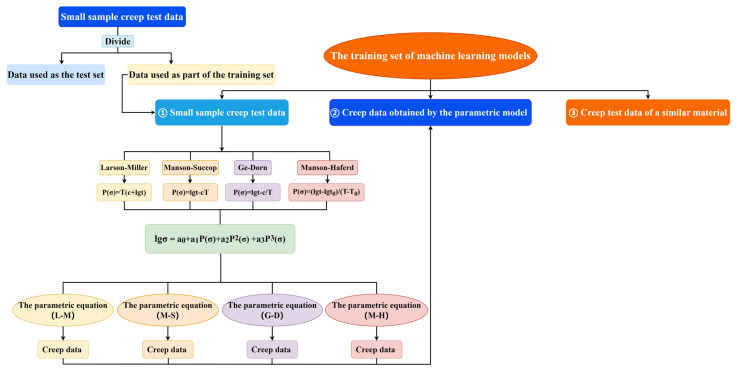
The schematic diagram of the method combining the machine learning models with the time–temperature parametric models.

For the new method of time–temperature parametric model + machine learning model, the basis for predicting the new method remains the machine learning model, and the new method connects different time–temperature parametric models with the machine learning model via parametric data expansion. Compared with the training data set of a single machine learning model, the training data set of the new method possesses an additional data type, namely, the creep data expanded using different time–temperature parametric models (L-M, M-S, G-D, M-H). As shown in Figure 5, in this new method, the training set data of machine learning models consists of three parts. The first part comprises the small-sample creep test data of the material. The second part consists of the creep test data of a material similar to the small-sample material. The third part is the data predicted by parametric equations obtained using four types of time–temperature parametric models. Then, these three types of data are used as the training data sets of machine learning models. Compared with the machine learning method or the time–temperature parametric method, the new approach proposed in this paper combines the time–temperature parametric method with machine learning, giving full play to the advantages of both methods.

The problem caused by the excessive extension of the third part of the data to the training set of machine learning models is that, with the increasing proportion of the data obtained from parametric equations in the training data, the training results of the machine learning model are constantly close to the predicted results of the time–temperature parametric curve.

As a result, machine learning models do not take advantage of the fact that they can train data from different materials together in order to increase the amount of data and more accurately find the relationship between inputs and output.

The time–temperature parametric model fits the small sample data through different theories and establishes the relationship between the temperature, stress, and creep rupture life of the material. The method we proposed involves introducing this relationship into the training set of the machine learning model. In addition to expanding the sample size of the training set by introducing similar material to help the machine learning model find the relationship between the temperature, stress, and creep rupture life of the small-sample material, the parametric model data extension method also provides more data for the machine learning model. This helps the machine learning model to establish the relationship between temperature, stress, and the life of small-sample materials more accurately.

#### 2.3.2. A New Prediction Method of Creep Rupture Life

Due to the different theories of four time–temperature parametric models, the prediction results differ, even for the same set of creep test data. As such, it is not guaranteed that one parametric model will always have the strongest prediction ability. Therefore, four different time–temperature parametric models are all used to improve the prediction accuracy and applicability of creep rupture life prediction.

**Figure 6:**
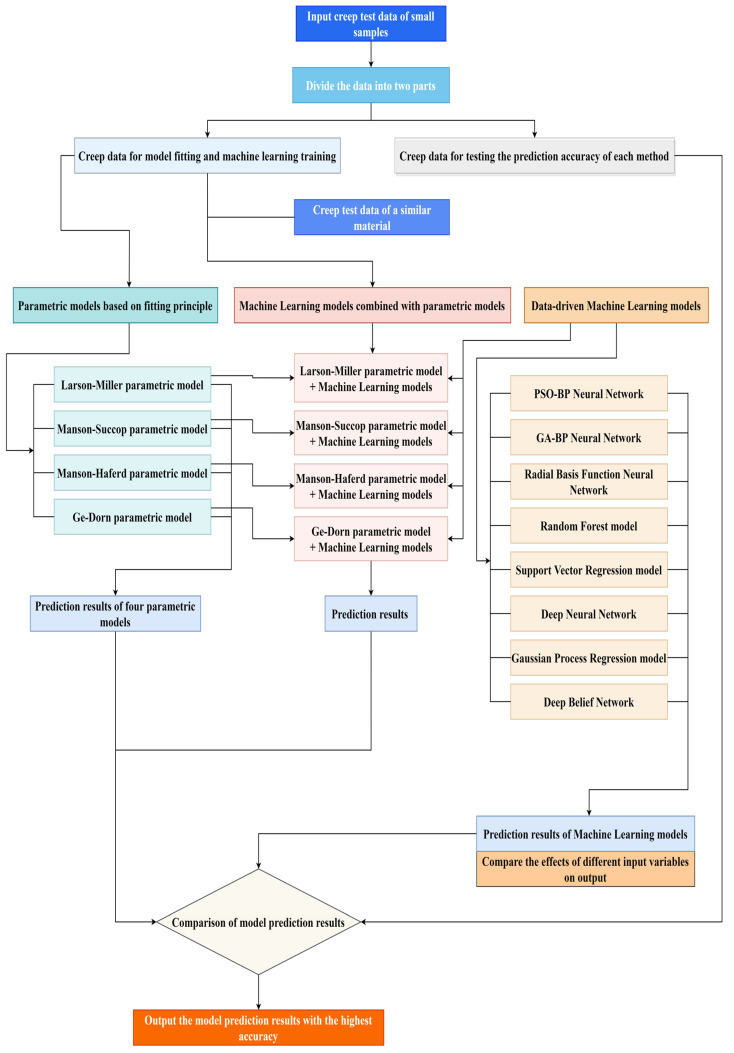
The flow diagram of a new creep rupture life prediction method.

When the sample size of the material is small, it is difficult to use machine learning models. Thus, it is necessary to add creep data for a material similar to small-sample material into the training set data of the machine learning models, and then use different machine learning models for training and prediction. Commonly used machine learning models include the random forest model, Gaussian process regression model, support vector regression model, and various neural network models (deep neural network model, deep belief network model, radial basis function, neural network model, etc.). Considering the differences in algorithmic theory between these machine learning models, the prediction results obtained after training with the same data are different. Therefore, the creep rupture life prediction method proposed in this paper simultaneously uses different machine learning models to obtain prediction results. Then, quantitative indicators (RMSE, R^2^, and MAPE) are calculated using predicted values and experimental values. As shown in Figure 6, the creep rupture life prediction method proposed in this paper utilizes different machine learning models to predict the creep rupture life of small-sample material. The prediction accuracy of each model is evaluated by comparing their quantitative indicators (RMSE, R^2^, and MAPE), and the output values of the model with the highest prediction accuracy are selected as the final output.

### 2.4. Indicators for Model Evaluation

(1) Root-Mean-Square Error

The root-mean-square error is the standard deviation of residuals. RMSE quantifies the degree of residual dispersion, revealing how tightly experimental values cluster around predicted values. This measures the deviation of predicted values from true values. The mathematical equation for RMSE is as follows:

(18) $$RMSE=\sqrt{\frac{1}{N}\sum_{i=0}^{N-1}{\left(y_{i}-{\hat{y}}_{i}\right)}^{2}}$$

where $y_{i}$ is the experimental values, ${\hat{y}}_{i}$ is the predicted values, and N is the number of experimental data.

(2) Mean Absolute Percentage Error

The mean absolute percentage error is an indicator used to measure the prediction accuracy of the model, reflecting the percentage difference between predicted values and experimental values. The smaller the MAPE, the higher the prediction accuracy of the model will be. The mathematical equation for MAPE is as follows:

(19) $$MAPE=\frac{1}{N}\sum_{i=0}^{N-1}\left|\frac{y_{i}-{\hat{y}}_{i}}{y_{i}}\right|$$

where $y_{i}$ is the experimental values, ${\hat{y}}_{i}$ is the predicted values, and N is the number of experimental data.

(3) Coefficient of determination

The coefficient of determination $R^{2}$ is used to characterize a good or bad fit based on the variation in data. From Equation (20), we know that the normal range of $R^{2}$ is [0, 1]. The closer the value of $R^{2}$ is to 1, the better the fitting effect of the model will be. The mathematical equation for $R^{2}$ is as follows:

(20) $$R^{2}=1-\frac{\sum_{i=0}^{N-1}{\left(y_{i}-{\hat{y}}_{i}\right)}^{2}}{\sum_{i=0}^{N-1}{\left(y_{i}-\bar{y}\right)}^{2}}$$

where $y_{i}$ is the experimental values, ${\hat{y}}_{i}$ is the predicted values, $\bar{y}$ is the mean of the experimental values, and N is the number of experimental data.

## 3. Results and Discussion

### 3.1. Establishment of Data Sets for Model Fitting and Training

We used 21 sets of creep test data from 5Cr-0.5Mo alloy standard plate specimens in the database of the National Institute of Materials Science as small-sample data, and the alloy number of 21 sets of data in the database was STBA25 [62]. Small-sample data were divided according to a ratio of approximately 7:3, in which 15 sets of creep test data were used to fit parametric curves of 4 different time–temperature parametric models and as training sets in machine learning models. The other 6 sets of creep test data were used as test sets in order to examine the prediction accuracy of each model. In total, 220 sets of creep test data of 1Cr-0.5Mo alloy standard plate specimens in the database were used as extended data of the training set of machine learning models. The alloy number of 220 sets of data in the database was SCMV2NT [62].

Partial creep test data of the machine learning training set are shown in Table 1. The training set of the machine learning model comprised 13 input variables and 1 output variable. The input variables were the mass fraction of different elements (C, Si, Mn, P, S, Ni, Cr, Mo, Cu, Al, N), test temperature T, and test stress σ, and the output variable was the material logarithmic creep fracture time lgt.

### 3.2. Model Prediction Results

#### 3.2.1. Prediction Results of Each Model in the New Method

**Figure 7:**
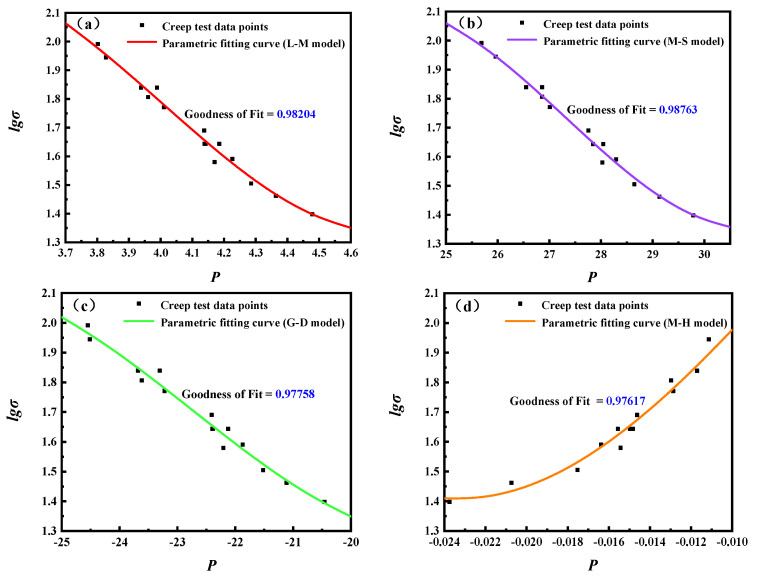
The fitting curves of four parametric models. (**a**) L-M model, (**b**) M-S model, (**c**) G-D model, (**d**) M-H model.

Three categories of methods were used to predict the creep rupture life of 5Cr-0.5Mo alloy. The first category of methods involved utilizing four kinds of time–temperature parametric models to fit 15 sets of creep test data from 5Cr-0.5Mo alloy through the L-M, M-S, G-D, and M-H models, respectively, and obtain four fitting curve functions for use in prediction. The fitting curves obtained using four kinds of parametric models are shown in Figure 7a–d. The values of coefficients and goodness of fitting curve functions are shown in Table 2.

The second category of methods uses different machine learning models for training and predicting, and the training set consists of two parts: 15 sets of creep data of 5Cr-0.5Mo alloy and 220 sets of creep data of 1Cr-0.5Mo alloy.

**Figure 8:**
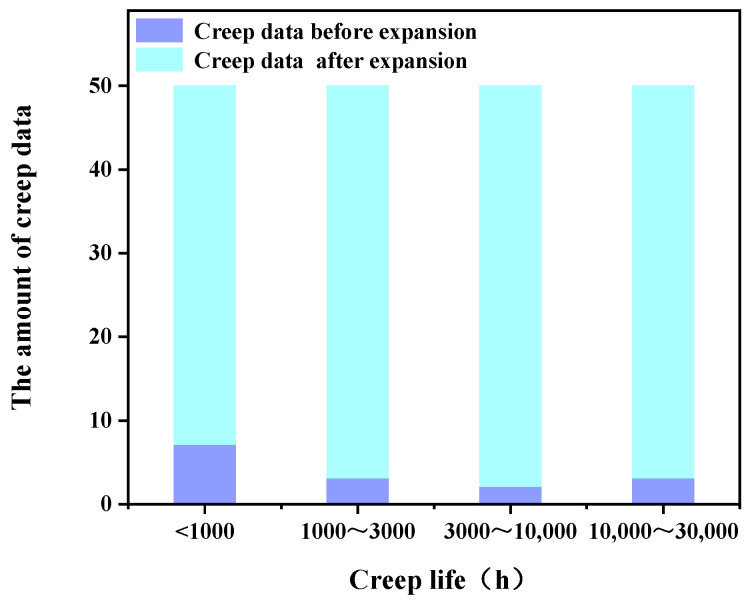
The comparison diagram of the creep data amount of various creep rupture life intervals before and after expansion.

The third category of methods combines the parametric models with the machine learning models, and the training set of machine learning models includes three parts of data: The first part is 15 sets of creep test data of 5Cr-0.5Mo alloy; the second part is the creep data predicted by the parametric equations obtained by fitting 15 stets of creep test data of 5Cr-0.5Mo alloy with different time–temperature parametric models. As shown in Figure 8, the creep data of various creep life intervals of 5Cr-0.5Mo alloy before expansion are 7 (<1000 h), 3 (1000~3000 h), 2 (3000~10,000 h), and 3 (10,000~30,000 h), respectively. After expansion, the creep data amount of each creep life interval is expanded to 50; the third part of the training set consists of 220 sets of creep test data of 1Cr-0.5Mo alloy.

**Figure 9:**
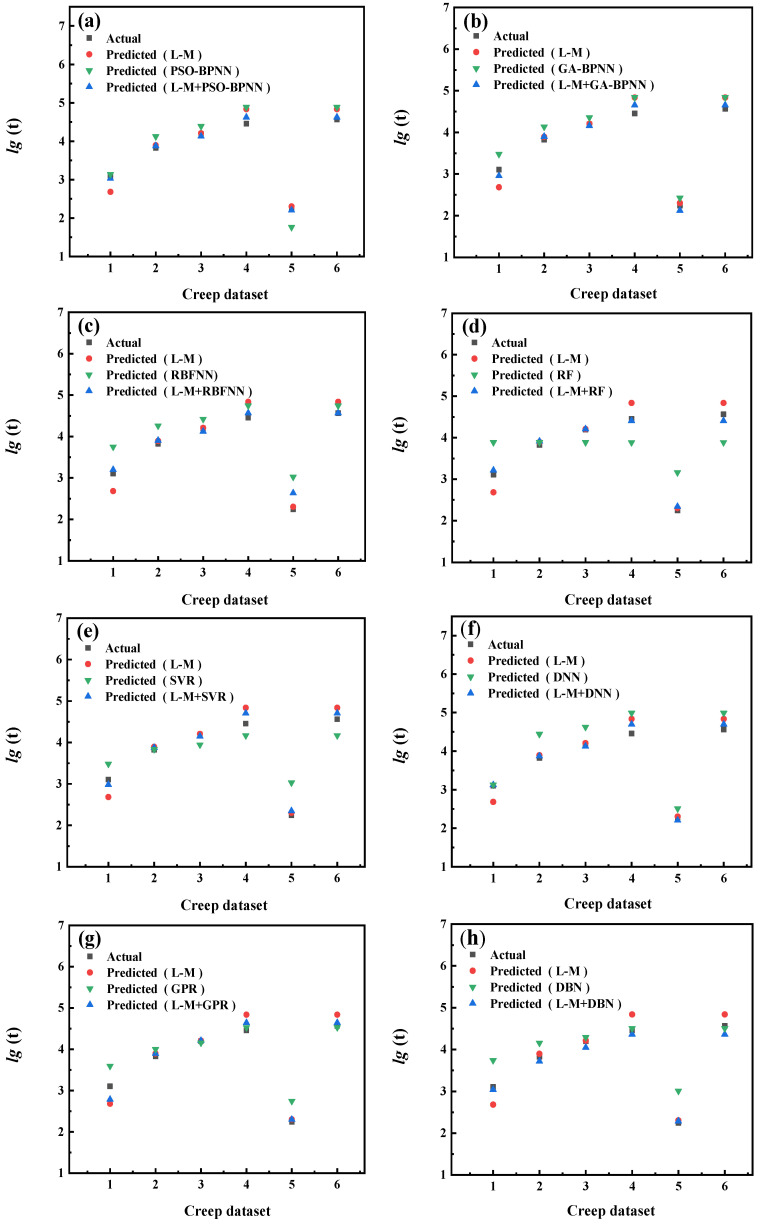
The comparison of prediction results between the L-M parametric model, machine learning models, and composite models. xxx (**a**) PSO−BPNN (**b**) GA−BPNN; (**c**) RBFNN; (**d**) RF; (**e**) SVR; (**f**) DNN; (**g**) GPR; (**h**) DBN.

**Figure 10:**
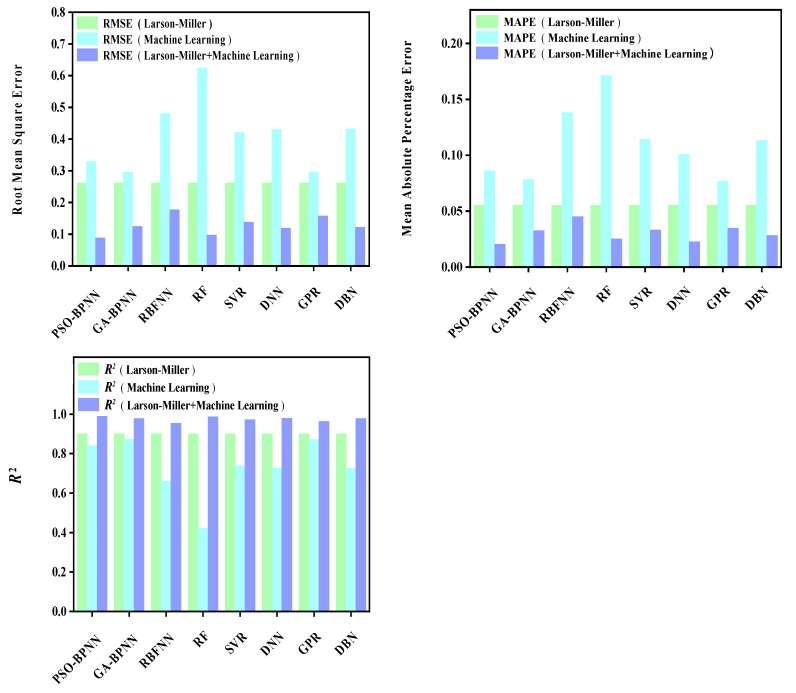
The values of three evaluation indicators of the L-M parametric model, machine learning models, and composite models.

**Figure 11:**
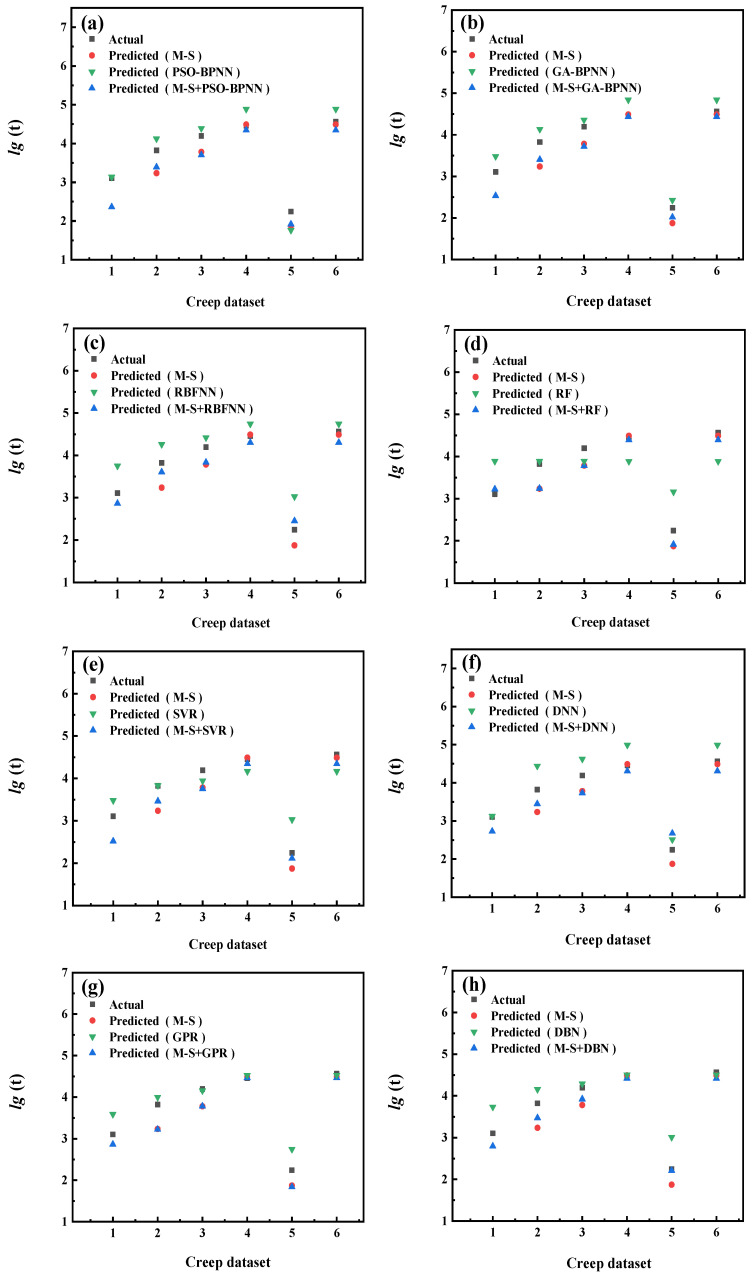
The comparison of prediction results between the M-S parametric model, machine learning models, and composite models. xxx (**a**) PSO−BPNN (**b**) GA−BPNN; (**c**) RBFNN; (**d**) RF; (**e**) SVR; (**f**) DNN; (**g**) GPR; (**h**) DBN.

**Figure 12:**
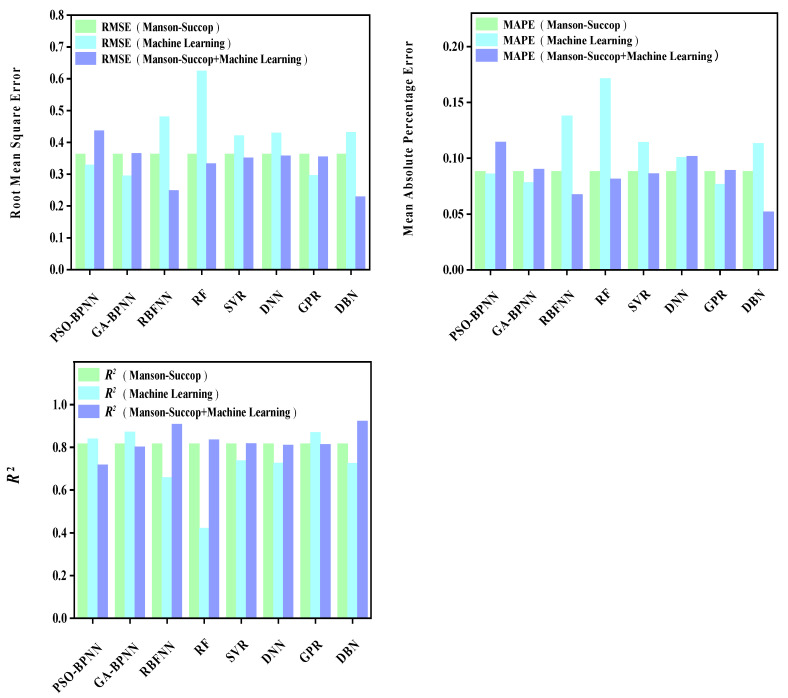
The values of three evaluation indicators of the M-S parametric model, machine learning models, and composite models.

**Figure 13:**
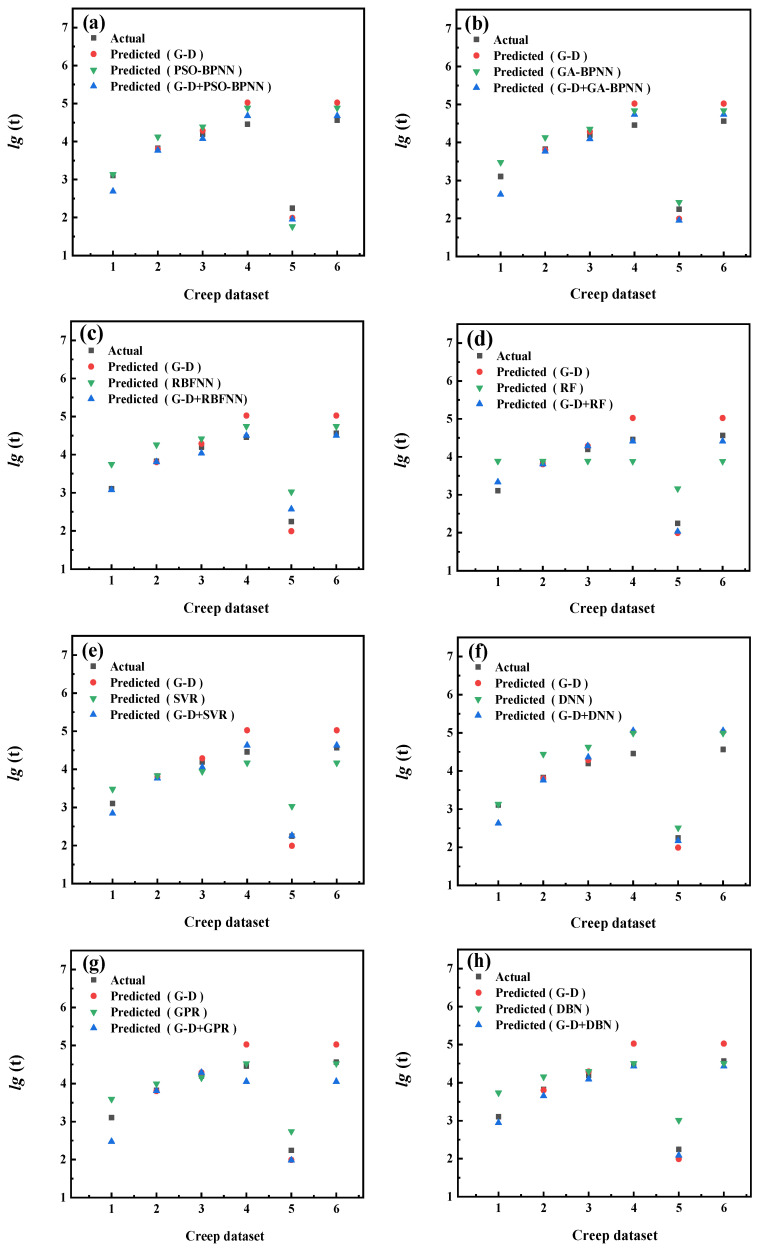
The comparison of prediction results between the G-D parametric model, machine learning models, and composite models. xxx (**a**) PSO−BPNN (**b**) GA−BPNN; (**c**) RBFNN; (**d**) RF; (**e**) SVR; (**f**) DNN; (**g**) GPR; (**h**) DBN.

**Figure 14:**
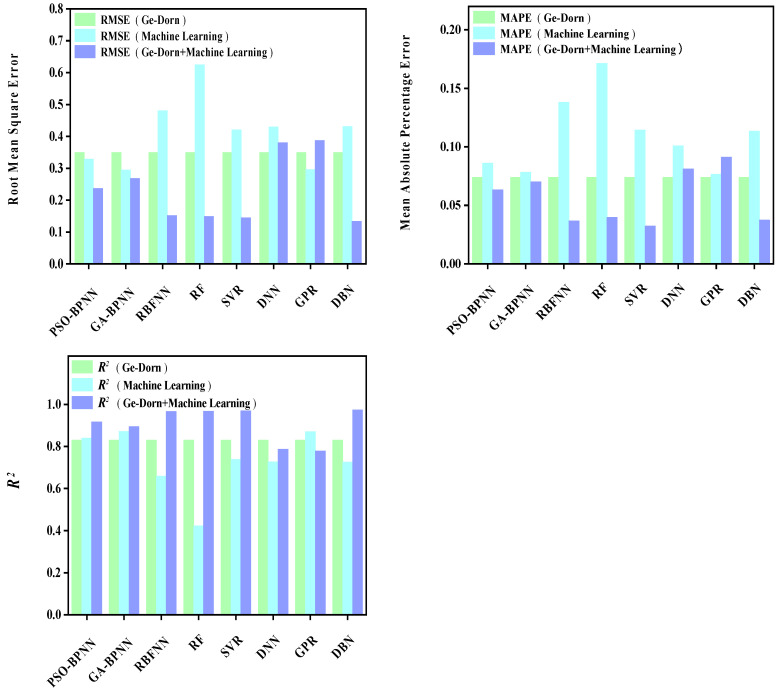
The values of three evaluation indicators of the G-D parametric model, machine learning models, and composite models.

**Figure 15:**
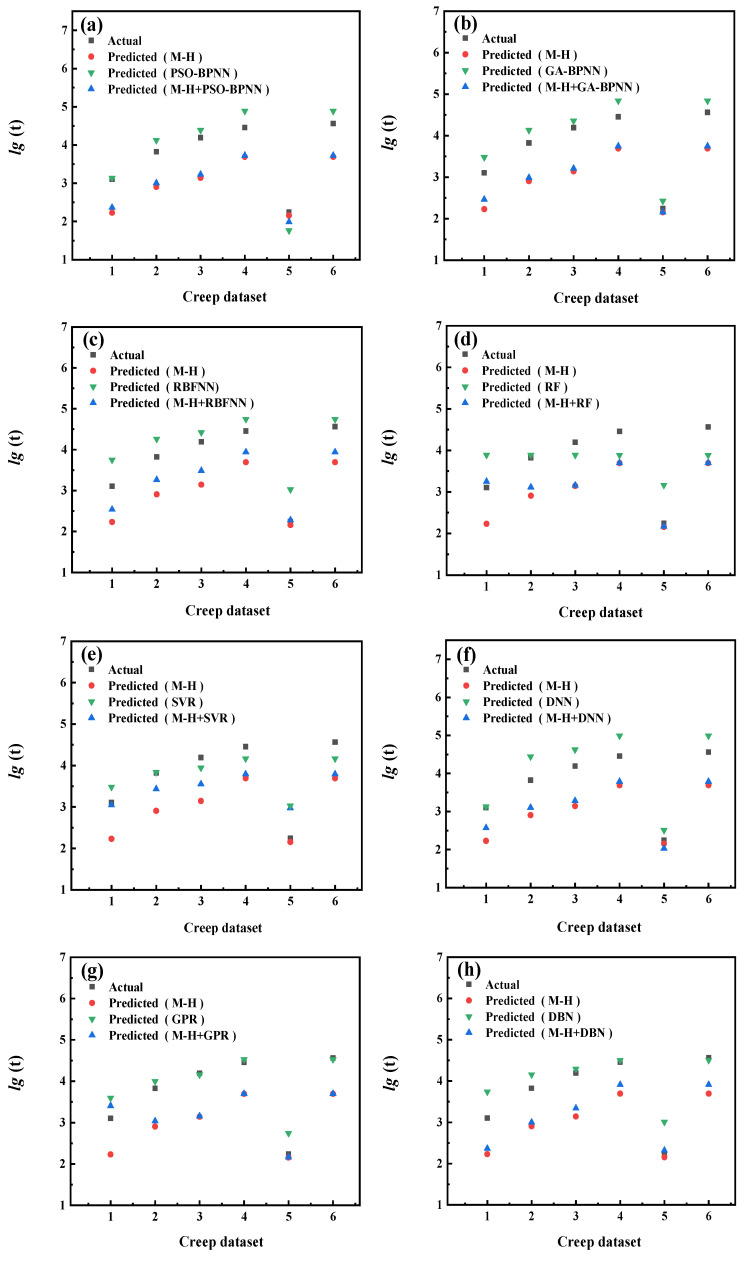
The comparison of prediction results between the M-H parametric model, machine learning models, and composite models. xxx (**a**) PSO−BPNN (**b**) GA−BPNN; (**c**) RBFNN; (**d**) RF; (**e**) SVR; (**f**) DNN; (**g**) GPR; (**h**) DBN.

**Figure 16:**
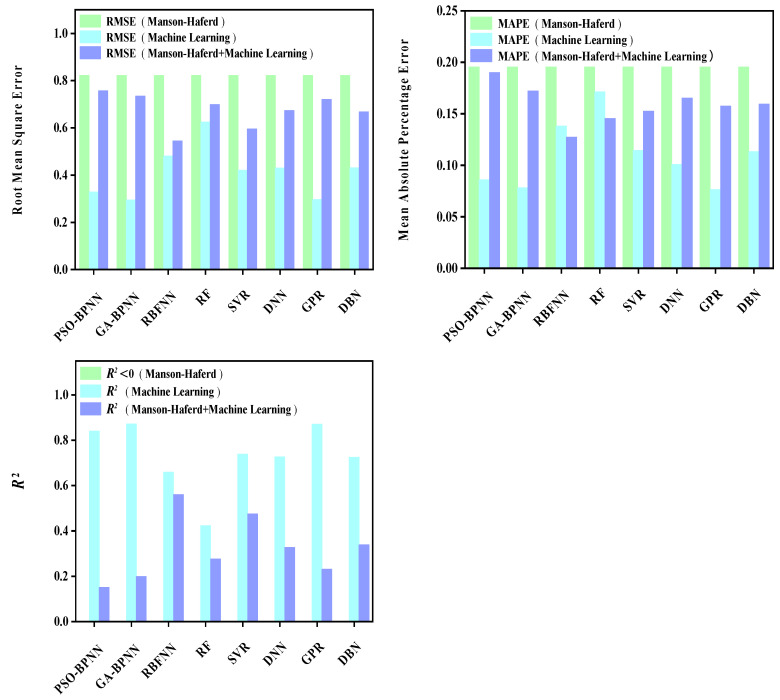
The values of three evaluation indicators of the M-H parametric model, machine learning models, and composite models.

The prediction results of each model are shown in Figure 9, Figure 10, Figure 11, Figure 12, Figure 13, Figure 14, Figure 15 and Figure 16, which show the actual test values and predicted values of three categories of methods. Three categories of methods in question include four time–temperature parametric models (L-M, M-S, G-D, M-H), eight machine learning models (PSO-BPNN, GA-BPNN, RBFNN, RF, SVR, DNN, GPR, DNN), and composite models ((L-M/M-S/G-D/M-H) + (PSO-BPNN/GA-BPNN/RBFNN/RF/SVR/DNN/GPR/DBN)).

Figure 9 shows the actual test values and the predicted values of three categories of methods (L-M model/machine learning models/L-M+ machine learning models). The L-M model uses 15 sets of creep test data of 5Cr-0.5Mo, the machine learning model uses creep test data of 5Cr-0.5Mo, and the creep test data of another material, 1Cr-0.5Mo. L-M+ machine learning models use three parts of data, namely, small-sample creep test data of 5Cr-0.5Mo, creep test data of 1Cr-0.5Mo, and creep data expanded using the L-M parametric model. The actual values shown in Figure 9 are another 6 sets of creep test data in 21 sets of 5Cr-0.5Mo.

After the predicted values of each model are obtained, three quantitative evaluation indicators are calculated for each model through the experimental values and predicted values. Figure 10 shows the values of three evaluation indicators of each model, namely, RMSE, MAPE, and R^2^. Figure 9 and Figure 10 show that, compared with the machine learning models, the model prediction results obtained by combining the L-M parametric model with various machine learning models are more accurate. Further, their predicted values are closer to those of actual test values.

The results show that the L-M parametric model helps the training process of the machine learning model to find the relationship between the input and output of the small-sample material more accurately. The combination of the L-M parametric model and the machine learning model improves the prediction accuracy of material creep rupture life.

Figure 11 shows the actual test values and predicted values of three categories of methods (M-S model/machine learning models/composite models). Figure 12 shows the values of three evaluation indicators of each model—RMSE, MAPE, and R^2^. Limited by the computational domain of the cubic fitting function of the M-S model, the M-S model cannot predict one test condition among the six test conditions in the set. The results reported in Figure 11 and Figure 12 show that, compared with the machine learning models, the model prediction results obtained by combining the M-S parametric model with some machine learning models are more accurate than others and their predicted values are closer to those of actual test values.

Figure 13 shows the actual test values and the predicted values of three categories of methods (G-D model/machine learning models/composite models). Figure 14 shows the values of three evaluation indicators of each model—RMSE, MAPE, and R^2^. Limited by the computational domain of the cubic fitting function of the G-D model, there is a test condition that the G-D model cannot predict among the six test conditions in the set. Figure 13 and Figure 14 show that, compared with the machine learning models, the model prediction results obtained by combining the G-D parametric model with most machine learning models become more accurate and their predicted values grow closer to those of the actual test values.

Figure 15 shows the actual test values and predicted values of three categories of methods (M-H model/machine learning models/composite models). Figure 16 shows the values of three evaluation indicators of each model—RMSE, MAPE, and R^2^. Figure 15 and Figure 16 show that, compared with the machine learning models, the accuracy of the model prediction results obtained by combining the M-H parametric model with various machine learning models is not significantly improved, which is caused by the low prediction accuracy of M-H parametric model for this set of creep data.

In summary, the creep rupture life of a small-sample material 5Cr-0.5Mo alloy can be predicted using time–temperature parametric models, machine learning models, and a new method combining time–temperature parametric models with machine learning models. The prediction results are compared, and the prediction accuracy of each model is quantified by three quantitative indicators (RMSE, MAPE, R^2^).

The results show that the prediction accuracy of 5Cr-0.5Mo alloy is improved by combining L-M, M-S, and G-D parametric models with various machine learning models. However, due to the low prediction accuracy of the M-H parametric model for this set of creep data, the prediction effect of combining machine learning models with an M-H parametric model is poor.

On the basis of the above phenomena, it can be seen that when a time–temperature parametric model is combined with machine learning models, the parametric model with the best prediction accuracy should be selected from the four time–temperature parametric models (L-M model, M-S model, G-D model, M-H model) in order to achieve a better combination effect.

#### 3.2.2. Comparison of Model Prediction Accuracy

We calculated the values of evaluation indicators for each model in the creep rupture life prediction system, and the results are shown in Table 3, which show the values of evaluation indicators RMSE, MAPE, and R^2^ of each model in three categories of methods (time–temperature parametric models/machine learning models/composite models).

From the statistical values shown in Table 3, it is possible to observe the L-M model with the highest prediction accuracy and the M-H model with the lowest prediction accuracy among the four time–temperature parametric models for this set of Cr-Mo alloy creep data. Among the eight machine learning models, the GA-BPNN model has the highest prediction accuracy. Among the 32 composite models, the L-M+PSO-BPNN composite model possesses the highest prediction accuracy. Furthermore, among these models of three categories of methods, the most accurate one is the L-M+PSO-BPNN composite model.

### 3.3. Comparison of Effects of Different Input Variables on Creep Rupture Life

Distinguished among the models of material creep rupture life prediction, compared with time–temperature parametric models, machine learning models have the unique advantage of quantifying the influence of various input variables on material creep rupture life.

The random forest model in machine learning models is an effective and frequently used method of quantifying the feature importance of various input variables to the output.

The influence of different input variables on creep life is compared by calculating feature importance scores in the random forest model of machine learning models. The feature importance evaluation method of the random forest model involves calculating the contribution of each feature in each decision tree of the model and then using the average value of the contribution to derive the importance of each feature to the output results.

**Figure 17:**
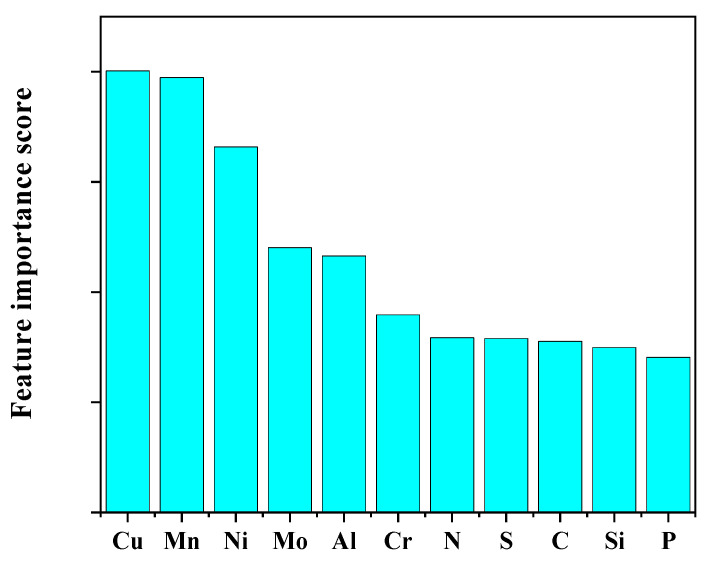
Feature importance scores of different input variables.

The influence of different input variables on the creep rupture life of 5Cr-0.5Mo and 1Cr-0.5Mo alloys is shown in Figure 17. It can be seen from Figure 17 that the top five alloy elements with high feature importance scores are Cu, Mn, Ni, Mo, and Al, indicating that the mass fraction content of these five elements has a more substantial effect on alloy creep rupture life than the other six elements.

## 4. Conclusions

(1) In this paper, a new creep rupture life prediction method is proposed that obtains the parametric equation of creep rupture life, stress, and temperature using four different time–temperature parametric models. Then, the creep rupture life data of other temperature and stress conditions predicted via parametric equations are used as the expansion of the training set data of various machine learning models. The new method combines the advanced machine learning models with the classical time–temperature parametric models. This measure not only solves the problem that the machine learning model is difficult to use for small samples but also improves the prediction accuracy of the machine learning model;
(2) Due to the different theories of various creep rupture life prediction models, the prediction results obtained using various prediction models are different, even for the same set of creep data. Additionally, the prediction abilities of models are variable, making it impossible to guarantee that a certain model will always have the strongest prediction ability for a variety of materials. Therefore, we propose a new creep rupture life prediction method in this paper that uses multiple models of three categories of methods simultaneously, compares the prediction accuracy of different models, outputs the predicted model values with the highest accuracy, and improves the prediction accuracy and applicability of the material creep rupture life prediction. The creep rupture life prediction method proposed in this paper can be further improved via the introduction of more machine learning models to further improve the prediction accuracy and applicability of the method;
(3) Compared with the classical parametric models (L-M, M-S, G-D, and M-H), the unique advantage of the machine learning model is that it can quantify the feature importance of different input variables. However, in the case of small-sample creep data, the prediction accuracy of machine learning models is often low, leading to the reliability of quantitative feature importance scores also being low. The new method proposed in this paper can improve the prediction accuracy of machine learning models in the case of small samples and quantify the influence of different input variables on output more accurately and reliably.

## Figures and Tables

**Table 1:** Partial creep test data of the machine learning training set.

Chemical Formula	T/°C	σ/MPa	Chemical Composition (wt.%)											lg(t)
			C	Si	Mn	P	S	Ni	Cr	Mo	Cu	Al	N	
5Cr-0.5Mo	550	88	0.1	0.27	0.45	0.014	0.006	0	4.31	0.59	0.1	0.002	0.0164	3.526080692
	550	64	0.1	0.27	0.45	0.014	0.006	0	4.31	0.59	0.1	0.002	0.0164	4.424718337
	600	98	0.1	0.27	0.45	0.014	0.006	0	4.31	0.59	0.1	0.002	0.0164	1.886490725
	600	69	0.1	0.27	0.45	0.014	0.006	0	4.31	0.59	0.1	0.002	0.0164	2.752816431
1Cr-0.5Mo	450	422	0.14	0.25	0.57	0.011	0.009	0.15	0.96	0.53	0.14	0.005	0.0098	2.256958153
	450	412	0.14	0.25	0.57	0.011	0.009	0.15	0.96	0.53	0.14	0.005	0.0098	2.682686478
	650	41	0.14	0.25	0.55	0.012	0.011	0.14	0.91	0.54	0.14	0.017	0.0098	2.831741834
	650	29	0.14	0.25	0.55	0.012	0.011	0.14	0.91	0.54	0.14	0.017	0.0098	3.530814194

**Table 2:** The values of coefficients and goodness of four fitting curve functions.

Model	Cubic Term (a_3_)	Quadratic Term (a_2_)	First Power Term (a_1_)	Constant Term (a_0_)	Goodness of Fit
L-M	0.73077384	−8.78500953	34.21754956	−41.28994736	0.98204
M-S	0.00475337475	−0.389710496	10.4841186	−90.7454929	0.98763
G-D	0.0031320327	0.213718871	4.70693983	35.0563393	0.97758
M-H	−73854.1678	−871.449158	78.5190039	2.7753578	0.97617

**Table 3:** The values of evaluation indicators of each model in the creep life prediction method.

The Category of the Model	Model	R-Squared	RMSE	MAPE
Time-temperature parametric models	L-M	0.89915	0.26079	0.05516
	M-S	0.81604	0.36274	0.08812
	G-D	0.82924	0.34949	0.07380
	M-H	−0.00229	0.82213	0.19542
Machine learning models	PSO-BPNN	0.83987	0.32860	0.08606
	GA-BPNN	0.87129	0.29462	0.07819
	RBFNN	0.65821	0.48009	0.13802
	RF	0.42197	0.62433	0.17128
	SVR	0.73761	0.42065	0.11426
	DNN	0.72609	0.42978	0.10071
	GPR	0.86998	0.29611	0.07653
	DBN	0.72438	0.43112	0.11329
Composite models	L-M + PSO-BPNN	0.98855	0.08786	0.02033
	L-M + GA-BPNN	0.97715	0.12413	0.03246
	L-M + RBFNN	0.95364	0.17682	0.04506
	L-M + RF	0.98608	0.09688	0.02512
	L-M + SVR	0.97179	0.13793	0.03308
	L-M + DNN	0.97902	0.11895	0.02249
	L-M + GPR	0.96317	0.15759	0.03465
	L-M + DBN	0.97797	0.12189	0.02823
	M-S + PSO-BPNN	0.71748	0.43648	0.11446
	M-S + GA-BPNN	0.80245	0.36499	0.09021
	M-S + RBFNN	0.90821	0.24880	0.06756
	M-S + RF	0.83555	0.33301	0.08134
	M-S + SVR	0.81723	0.35107	0.08621
	M-S + DNN	0.81055	0.35743	0.10176
	M-S + GPR	0.81342	0.35471	0.08910
	M-S + DBN	0.92224	0.22899	0.05187
	G-D + PSO-BPNN	0.91711	0.23642	0.06321
	G-D + GA-BPNN	0.89371	0.26772	0.07017
	G-D + RBFNN	0.96582	0.15182	0.03663
	G-D + RF	0.96735	0.14838	0.03966
	G-D + SVR	0.96902	0.14453	0.03223
	G-D + DNN	0.78624	0.37967	0.08100
	G-D + GPR	0.77820	0.38675	0.09104
	G-D + DBN	0.97352	0.13364	0.03737
	M-H + PSO-BPNN	0.15121	0.75656	0.19010
	M-H + GA-BPNN	0.19909	0.73491	0.17209
	M-H + RBFNN	0.56002	0.54470	0.12729
	M-H + RF	0.27659	0.69845	0.14541
	M-H + SVR	0.47376	0.59571	0.15259
	M-H + DNN	0.32738	0.67349	0.16517
	M-H + GPR	0.23094	0.72015	0.15742
	M-H + DBN	0.33878	0.66775	0.15937

## Data Availability

Not applicable.
